# Diet Supplementation With Sulfur Amino Acids Modulated Fermentation Metabolome and Gut Microbiome in Goats

**DOI:** 10.3389/fmicb.2022.870385

**Published:** 2022-05-24

**Authors:** Tsegay Teklebrhan, Zhiliang Tan

**Affiliations:** ^1^CAS Key Laboratory for Agro-Ecological Processes in Subtropical Region, National Engineering Laboratory for Pollution Control and Waste Utilization in Livestock and Poultry Production, Institute of Subtropical Agriculture, The Chinese Academy of Sciences, Changsha, China; ^2^University of Chinese Academy of Sciences, Beijing, China; ^3^School of Animal and Range Sciences, Haramaya University, Dire Dawa, Ethiopia

**Keywords:** amino acids, hydrogen, metabolites, metagenomics, microbiome

## Abstract

Dietary amino acids shift hydrogen metabolism to an alternative hydrogen sink consisting of dissolved hydrogen sulfur (dH_2_S) rather than methanogenesis; and influences the fermentation metabolome and microbiome associated with particles and liquid fractions in gut regions (foregut, small intestine, and hindgut) of goats. A completely randomized block design with a total of 20 goats (5 goats per treatment) was used to conduct the trial. The goats were fed on a diet that consisted of a concentrated mixture with maize stover roughage (50:50, on a dry matter basis) and randomly assigned to one of the four treatments: without amino acid supplementation (a basal diet), a basal diet supplemented with methionine (Met), a basal diet supplemented with lysine (Lys), and a basal diet supplemented with methionine and lysine (ML). Goats fed Met alone or in combination had less acetate, acetate to propionate ratio, and greater propionate (*p* < 0.05) in the foregut and hindgut than those fed control or Lys. Nonetheless, the goats fed on the amino acid supplements had higher levels of branched-chain VFA (*p* < 0.05) in the foregut and hindgut than the control goats. Goats fed on ML had the highest ammonia (*p* < 0.01), followed by Met or Lys, both in the foregut and hindgut, compared with the control. Those fed on Met alone or in combination, had lower dH_2_, dCH_4_ (*p* < 0.01), and higher dH_2_S (*p* < 0.01) in the foregut and hindgut than the control or Lys. The goats that were fed on Met alone or in combination, had higher 16S rRNA gene copies of total bacteria, methanogens, and 18S rRNA gene copies of protozoa, fungi, and fiber-utilizing bacterial species (*p* < 0.01) associated with particles vs. liquid, both in the foregut and hindgut than the control goats. This study gives insights into the use of sulfur-containing amino acids, as an alternative dietary mitigation strategy of methanogenesis in ruminants and highlights the need for further research in this direction.

## Introduction

Microbial fermentation of amino acids produces ammonia, volatile fatty acids (VFA), carbon dioxide (CO_2_), methane (CH_4_), and molecular hydrogen (H_2_) in the gut (foregut, small intestine, and hindgut) of ruminants. It is crucial to reduce the level of crude protein (CP) in ruminant diets with the supplementation of limiting amino acids such as methionine and lysine, to improve the number of metabolizable amino acids; and decrease nitrogen losses, feed costs, and greenhouse gas emissions without adverse effect on animal performance (Sinclair, 2014; Guyader et al., 2016). It has been shown that dietary supplements with high sulfate could shift H_2_ toward energetically advantageous pathways away from methanogenesis and reduce methane emissions and yield (Judy et al., 2019; Lan and Yang, 2019; Teklebrhan et al., 2020). For instance, a sulfur-rich corn gluten diet decreased dissolved hydrogen (dH_2_) and methane (dCH_4_), while increasing dissolved hydrogen sulfur (dH_2_S) which was associated with reduced methanogenesis in goats fed on corn meal (Teklebrhan et al., 2020). Hydrogen has been shown to be involved in amino acid fermentation in several ways. In some cases, hydrogen or reducing equivalents required for hydrogenation reactions can be obtained by the uptake of molecular hydrogen or may be generated from one amino acid for the reduction of another (Nisman, 1954; Barker, 1961). This suggests that amino acid biosynthesis can either release or consume H_2_, which could affect gut fermentation pathways and gaseous production. Biosynthesis of sulfur-containing amino acids like methionine involves H_2_ being reduced to H_2_S, suggesting that methionine biosynthesis could facilitate uptake of H_2_ by sulfidogenic bacteria rather than methanogens.

Research on diet supplementation with amino acids is limited to using amino acids in microbial fermentation and microbiome patterns in ruminants, though few *in vitro* trials Abbasi et al. (2019) and Hassan et al. (2021) reported inconsistent results for lysine and methionine in fermentation and microbiota. Hence, it is crucial to investigate and compare the effects of supplementation of dietary methionine or lysine, either alone or in combination with a low protein diet in modulating microbial fermentation and the microbial ecosystem in the gut (foregut, small intestine, and hindgut) of ruminants. We hypothesized that methionine supplementation, either alone or combination in corn stover based diet could shift hydrogen metabolism toward an alternative electron sink and modulates fermentation metabolome and microbiome in goats. As a result, this study investigated that sulfur amino acids shift hydrogen to H_2_S than methanogenesis and altered the microbiome associated with solid and liquid fractions in the gut regions of goats.

## Materials and Methods

### Animals Feeding and Management

The study used twenty Liuyang black male goats with an average age of 10 ± 0.2 months old and an initial body weight of 18.2 ± 2.5 kg. The experiment was conducted using a completely randomized block design with a total of 20 goats (5 goats per treatment). All of the goats were kept in stainless steel metabolic cages (150 cm × 60 cm × 80 cm) with free access to clean water. The metabolic room’s temperature was set to 22 ± 1°C. The diet was designed to meet 140% of the metabolic energy maintenance needs (Lu and Zhang, 1996). The ingredients and nutrient composition of a basic diet are given in Table 1. In total, twenty goats were randomly divided into four groups. Each group of five goats was randomly assigned to one of four diets: a basal diet with no amino acid supplementation (control), a control diet supplemented with methionine (Met), a control diet supplemented with lysine (Lys), and a control diet supplemented with both methionine and lysine (ML). Goats were adapted to treatment diets through step-wise increments for 14 days until they all reached their stable dry matter (DM) intake according to the standard of metabolic body weight. The experimental period lasted for another 12 d. Goats were fed *ad libitum*, targeting less than 5% refusal. Daily meals were offered twice equally at 8:00 am and 4:00 am. The amounts of methionine and lysine supplement in Met, Lys, and ML treatments were 1.27 g, 1.96 g, and 1.27 plus 1.96 g of concentrate on a DM basis, respectively, according to Sun et al. (2007). Briefly, the amounts of supplements were computed from the intestinal degradability of amino acids and the pattern in muscle protein of goats in order to balance the supply of amino acids to the duodenum. To balance the extra effect of nitrogen from the amino acid supplements, control, Met, and Lys were supplemented with 1.08, 0.81, and 0.25 g urea per 100 g of concentrate, respectively. The duodenal and ileal flows, as well as ileal apparent digestibility of amino acids, were calculated according to the following equations:


(1) $$Q_{d}=\frac{C_{t}}{C_{d}}$$

(2) $$Q_{i}=\frac{C_{t}}{C_{i}}$$

(3) $$D\,F\,A\,A_{i}=C\,D\,A\,A_{i}\times Q_{d}$$

(4) $$I\,F\,A\,A_{i}=C\,I\,A\,A_{i}\times Q_{i}$$

(5) $$D\,A\,A_{i}=\frac{D\,F\,A\,A_{i}-I\,F\,A\,A_{i}}{D\,F\,A\,A_{i}}\times 100\%$$

**TABLE 1:** Ingredients^a^ and nutrient composition of the diet.

Item	Basal diet
Ingredient (g/kg DM)	
Corn	224
Wheat bran	179
Soybean meal	60.0
Rapeseed meal	1.00
Maize straw	500
Urea	10.8
Salt	6.00
Vitamin/mineral premix^b^	20.0
Nutrient composition (g/kg DM)	
Crude protein	126.9
Acid detergent fiber	231.8
Neutral detergent fiber	493.5
Calcium	2.00
Phosphorus	4.00
Metabolizable energy (MJ/kg)	2.26

*^a^Ingredients composition (% DM), contained 22.4% of corn, 17.9% wheat bran, 6.0% of soybean meal, 0,1% of rape seed meal, 50% of maize stover, 1.08% of urea, 0.6% of salt, and 2.0% of premix.*

*^b^Premix formulated (per kg of dietary DM): 119 g of MgSO_4_⋅H_2_O, 1.53 g of FeSO_4_⋅H_2_O, 0.8 g of CuSO_4_⋅5H_2_O, 3 g of MnSO_4_⋅H_2_O, 5 g of ZnSO_4_⋅H_2_O, 10 mg of Na_2_SeO_3_, 40 mg of KI, 30 mg of CoCl_2_⋅6H_2_O, 95,000 IU of vitamin A, 17,500 IU of vitamin D, and 18,000 IU of vitamin E.*

where Q_d_ is the DM flow in the duodenum, C_t_ the total amount of the administrated Cr_2_O_3_ in the rumen per day, C_d_ the Cr_2_O_3_ content in the dried duodenal digesta, Q_i_ the DM flow at the ileum, C_i_ the Cr_2_O_3_ content in the dried ileal digesta, DFAA_i_ the AA_i_ flow at the duodenum, CDAA_i_ the AA_i_ content in the dried duodenal digesta, IFAA_i_ the AAi flow at the ileum, CIAA_i_ the AA_i_ content in the dried ileal digesta, and DAA_i_ is the ileal apparent digestibility of AAi. In addition, the amounts of Met and Lys infused into the lumen of the duodenum were calculated by the following equation as follows:


(6) $$\frac{D\,A\,A_{i}}{R_{i}}\,△\,X_{i}-\sum_{i=1}^{n}D\,A\,A_{i}\times △\,X_{i}=D_{t}\,Q_{t}-\frac{D_{i}\times D\,F\,A\,A_{i}}{R_{i}}$$

where D_t_ is the ileal digestibility of total amino acids, Q_t_ is the duodenal flow of total amino acids, ΔX_i_ is the computed amount of AA_i_ infused into the duodenum and R_i_ is the AA_i_ proportion of total amino acids in the muscle.

### Sampling and Processing

After the morning feed on d 12, 5 goats from each treatment were euthanized according to the ethical procedure of the Institute of Subtropical Agriculture, Chinese Academy of Sciences (procedure number: ISA-W-201802). The abdomen was opened, and the gut was immediately separated from the carcass. To avoid mixing of digesta for sampling, the gut regions (foregut, small intestine, and hindgut), including reticulo-rumen (foregut), duodenum, ileum, jejunum (small intestine), cecum, colon, and rectum (hindgut), were tied with a sterile thread at the start and end of each region. Each gut region was longitudinally incised along the dorsal line using sterile equipment. The contents in each gut region were first homogenized and then mixed thoroughly to reduce the localized effect.

**FIGURE 1:**
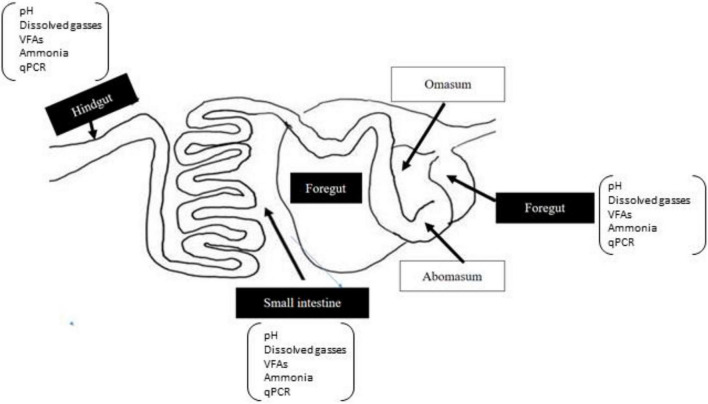
A schematic diagram of gut regions of goat. The sampled regions of the gut are highlighted in black boxes.

Representative samples of the foregut (∼100 g), small intestine (∼60 g), and hindgut (∼60 g) were collected in sterile anoxic tubes. A schematized diagram of the sampling regions is given in Figure 1. Approximately, 10 g of a subsample from each gut region was used for immediate measurement of dH_2_, dH_2_S, and pH. Another subsample of each gut region was diluted with 1:5 (m/v) iced sterile anaerobic phosphate-buffered saline (PBS; pH 6.8). Samples were then homogenized and filtered through four layers of sterile cheesecloth to obtain approximately 100 ml of liquid and remaining particle-associated samples from each region, respectively, for the liquid and particle-associated samples. Then samples were immediately snap-frozen using liquid nitrogen at −80°C for genomic DNA isolation. The remaining liquid from each region was used for the analysis of VFA, ammonia, and dCH_4_.

### Sample Analysis

All samples of the feed offered, and refusals were dried at 105°C for 24 h for DM determination and then ground, using a hammer mill to pass through a 1-mm sieve. Crude protein (CP) (N × 6.25) was determined using the Kjeldahl method (AOAC, 1995). Neutral detergent fiber (NDF) with the addition of α-amylase and sodium sulfite and acid detergent fiber (ADF), both expressed inclusive of residual ash, were analyzed according to Van Soest et al. (1991). Metabolizable energy (ME) was calculated, according to Lu and Zhang (1996). Phosphorus and Ca concentrations were determined, following the procedure of AOAC International (2006).

The pH of gut samples was measured using a portable pH meter (Starter 300; Ohaus Instruments Co., Ltd., Shanghai, China). The dH_2_ and dH_2_S of gut samples were determined by micro-sensor, using H_2_ and H_2_S electrodes, respectively, according to protocols of the manufacturer’s manual (Unisense, Aarhus, Denmark). Dissolved methane (dCH_4_) was extracted from the liquid phase of gut samples into the gas phase using the procedure of Wang et al. (2016a) with slight modification. A 20-ml syringe containing 10 ml of N_2_ gas (>99.99%) was transferred into a 50-ml plastic syringe containing 35 ml of gut samples *via* polyurethane tubing. The gas dissolved in the liquid phase was released into the gas phase, by shaking at 200 revolutions per second for 5 min in an orbital shaker (WSZ-100A, Shanghai Yiheng Scientific Instruments Co., Ltd., Shanghai, China). Gas samples were collected, using evacuated tubes for analysis, using GLC (Agilent 7890A, Agilent Inc., Palo Alto, CA, United States). Then, the concentration of dCH_4_ in the original liquid fraction was computed using the equation of Wang et al. (2016a). Frozen liquids were thawed and centrifuged at 12,000 × *g* for 10 min at 4°C, and supernatants were used to determine the VFA profile according to Wang et al. (2016a) using gas chromatography (Agilent 7890A, Agilent Inc., Palo Alto, CA, United States). Ammonia concentrations were measured according to Weatherburn (1967).

One ml liquid and 1 g particle-associated samples from each gut region were used for the isolation of genomic DNA following the procedures of Yu and Morrison (2004). The quality of the DNA was determined, using agarose gel electrophoresis (0.8%). The quantity of DNA was measured, using a NanoDrop ND-1000 spectrophotometer (NanoDrop Technologies Inc., Wilmington, DE, United States). The qPCR analysis of target microbes was performed, using an ABI 7900HT, Fast Real-Time PCR system (Applied Biosystems, Foster City, CA, United States) using SYBR Premix Ex Taq (Perfect Real Time, Takara, Shiga, Japan). A standard curve was generated for each bacterial species and total bacteria, methanogens, protozoa, and fungi, using plasmid DNA containing the insert for the primers as shown in Table 2. The ten-fold serial dilutions of each standard were made in RNase-free water for qPCR analysis. The qPCR reaction volume was 10 μl, including 5 μl of SYBR Premix Ex Taq, 0.2 μl of ROX, 0.2 μl of each primer (10 μ*M*), 1 μl of the template DNA (10 ng/μl), and 3.4 μl of RNase-free water. The program was set to 95°C for 30 s, followed by 40 cycles at 95°C for 5 s and 60°C for 30 s for annealing/extension. The final melting curve was detected at 95°C for 15 s, 60°C for 1 min, and 95°C for 15 s. The final absolute amount of the target group or species was estimated, by relating the cycle threshold (CT) value to the standard curves. The results were then transformed into log_10_ copies/ml or g of sample for further analysis.

**TABLE 2:** Primers are used for quantitative PCR (qPCR).

Target species	Primer	Primer sequence (5′-3′)	Size (bp)	Reference	E^1^ (%)
Bacteria	Forward	CGGCAACGAGCGCAACCC	146	Denman and McSweeney, 2006	100.4
	Reverse	CCATTGTAGCACGTGTGTAGCC			
Protozoa	Forward	GCTTTCGWTGGTAGTGTATT	223	Sylvester et al., 2004	96.3
	Reverse	CTTGCCCTCYAATCGTWCT			
Methanogens	Forward	GGATTAGATACCCSGGTAGT	192	Hook et al., 2009	101.9
	Reverse	GTTGARTCCAATTAAACCGCA			
Fungi	Forward	GAGGAAGTAAAAGTCGTAACAAGGTTTC	120	Denman and McSweeney, 2006	97.2
	Reverse	CAAATTCACAAAGGGTAGGATGATT			
*Prevotella ruminicola*	Forward	GAAAGTCGGATTAATGCTCTATGTTG	74	Stevenson and Weimer, 2007	99.8
	Reverse	CATCCTATAGCGGTAAACCTTTGG			
*Selenomonas ruminantium*	Forward	CAATAAGCATTCCGCCTGGG	138	Stevenson and Weimer, 2007	102.2
	Reverse	TTCACTCAATGTCAAGCCCTGG			
*Ruminococcus albus*	Forward	CCCTAAAAGCAGTCTTAGTTCG	176	Koike and Kobayashi, 2001	101.3
	Reverse	CCTCCTTGCGGTTAGAACA			
*Ruminococcus flavefaciens*	Forward	CGAACGGAGATAATTTGAGTTTACTTAGG	132	Denman and McSweeney, 2006	102.2
	Reverse	CGGTCTCTGTATGTTATGAGGTATTACC			
*Fibrobacter succinogenes*	Forward	GTTCGGAATTACTGGGCGTAAA	121	Denman and McSweeney, 2006	100.7
	Reverse	CGCCTGCCCCTGAACTATC			
*Ruminobacter amylophilus*	Forward	CTGGGGAGCTGCCTGAATG	102	Stevenson and Weimer, 2007	100.3
	Reverse	GCATCTGAATGCGACTGGTTG			

*^1^Efficiency.*

### Statistical Analysis

Fermentation metabolites and qPCR data were analyzed using the R software version 3.6.3 by the ([Bibr B36][Bibr B36], Vienna, Austria). Data were subjected to a linear mixed model, using the package lme4 version as described by Pinheiro et al. (2013). Including diet, gut regions, sample fraction, and all possible interactions between them, as fixed effects, and block or animal as a random effect. Multiple mean comparisons were tested, using Tukey’s adjustment. Differences at *p* ≤ 0.05 were considered significant results. Pearson correlation coefficients were computed to determine the correlations between fermentation metabolites and microbiota concentrations. Correlation coefficient values (*r*) with *r* > 0.44 for *p* < 0.1, *r* > 0.52, *p* < 0.05, *r* > 0.66 for *p* < 0.01, and *r* > 0.79 for *p* < 0.001. Correlation coefficient values greater than zero indicate a positive correlation while values less than zero indicate a negative correlation between variables.

## Results

### Short-Chain Fatty Acid Metabolites

Amino acid supplementation changed the production of volatile fatty acids in gut regions (Table 3). The highest total VFA production was obtained (+73 and 64%) in the foregut, followed by the hindgut and the small intestine, with a lower (−24%; *p* < 0.05) in the hindgut vs. foregut filtrates. The foregut had a higher acetate molar percentage and acetate to propionate ratio (*p* < 0.05), than the hindgut and small intestine filtrates. Nevertheless, propionate, valerate, and branched-chain VFA (isobutyrate and isovalerate) (*p* < 0.05) followed the reverse trend; being higher in the small intestine > hindgut > foregut. Except in the small intestine, goats fed Met alone or in combination (ML), had lower acetate, and acetate to propionate ratios, but higher propionate (*p* < 0.05) than those fed control or Lys. Nevertheless, goats fed on the amino acid supplements had greater branched-chain VFA (*p* < 0.05) than those in the control group. Individual fatty acids were modulated by the interaction of the gut with diet: goats fed Met alone or in combination had less acetate, and acetate to propionate ratio while; having more propionate (*p* < 0.05) in the foregut and hindgut than those fed control or Lys. In addition, consistently, greater branched-chain VFA (*p* < 0.01) was apparent; both in the foregut and hindgut of goats fed the amino acid supplements than in the control group. Despite the small intestine having the highest propionate and branched-chain VFA (*p* < 0.05), these values remained unaffected by the diet groups.

**TABLE 3:** Fatty acid metabolites in gut regions of goats supplemented with amino acid.

Gut regions	Diet^2^				Items				
		VFA^1^	Acetate	Propionate	Butyrate	Valerate	Isobutyrate	Isovalerate	Ace/prop
Small intestine	Control	18.1	42.1^c^	31.0^a^	14.0	4.00	4.20^a^	4.70^a^	1.35^c^
	Met	17.2	42.1^c^	30.1^a^	15.9	3.98	3.94^a^	3.98^a^	1.39^c^
	Lys	18.0	41.2^c^	32.6^a^	14.1	4.10	3.96^a^	4.04^a^	1.26^c^
	ML	19.3	42.0^c^	31.3^a^	13.0	4.70	4.64^a^	4.36^a^	1.34^c^
Foregut	Control	66.7	65.6^a^	16.0^c^	14.0	2.12	1.53^c^	0.75^c^	4.10^a^
	Met	67.5	54.9^b^	22.5^b^	14.9	2.16	2.67^b^	2.87^b^	2.44^b^
	Lys	68.3	63.6^a^	15.2^c^	14.1	1.99	2.62^b^	2.49^b^	4.18^a^
	ML	69.5	53.9^b^	23.5^b^	15.0	2.20	2.89^b^	2.51^b^	2.29^b^
Hindgut	Control	49.8	62.5^a^	19.1^c^	14.5	2.02	0.98^c^	0.90^c^	3.27^a^
	Met	50.4	51.8^b^	26.3^b^	14.8	2.32	2.90^b^	1.88^b^	1.97^b^
	Lys	51.3	61.5^a^	17.7^c^	13.9	2.03	2.46^b^	2.41^b^	3.47^a^
	ML	52.1	51.9^b^	25.7^b^	15.0	2.71	2.57^b^	2.12^b^	2.02^b^
	SEM	5.01	3.67	2.50	1.46	0.20	0.01	0.02	0.03
*P-value*	Gut	0.0201	0.0301	0.0401	0.1524	0.0201	0.0400	0.0300	0.0302
	Diet	0.1210	0.0400	0.0200	0.1310	0.1124	0.0201	0.0412	0.0200
	Gut*Diet	0.3212	0.0301	0.0410	0.2435	0.2010	0.0302	0.0402	0.0310

*^1^VFA total volatile fatty acids (mM), individual volatile fatty acids (mol/100 mol) acetate to propionate ratio (mol/mol), ^2^basic diet without supplementation (Control), control supplemented with methionine (Met), and control supplemented with lysine (Lys), and control supplemented with methionine and lysine (ML). Results beard different letters indicate significant, while results beard same letters indicate not significant variation.*

### Dissolved Gas Products

**FIGURE 2:**
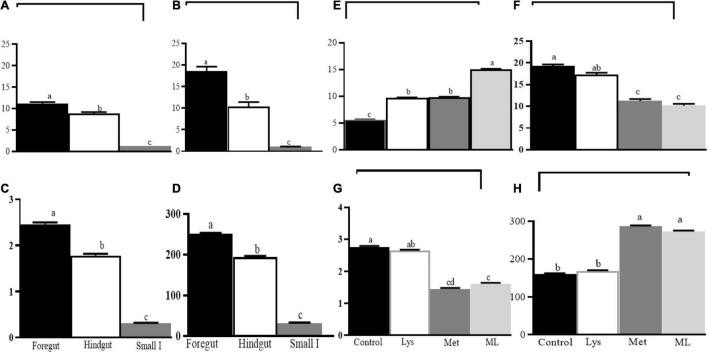
Least square and standard error of means (SEM) for the effect of gut and dietary amino acids, respectively, on microbial fermentation metabolome **(A)** ammonia production (mM), **(B)** dissolved hydrogen (dH_2_; μM), **(C)** dissolved methane (dCH_4_; mM), **(D)** dissolved hydrogen sulfur (dH_2_S; mM), and **(E)** ammonia production (mM), **(F)** dissolved hydrogen (dH_2_; μM), **(G)** dissolved methane (dCH_4_; mM), **(H)** dissolved hydrogen sulfur (dH_2_S; mM). Different letters on the top of bars for the gut reigns and, amino acid supplement, respectively, indicate significantly different at *p* < 0.05.

**FIGURE 3:**
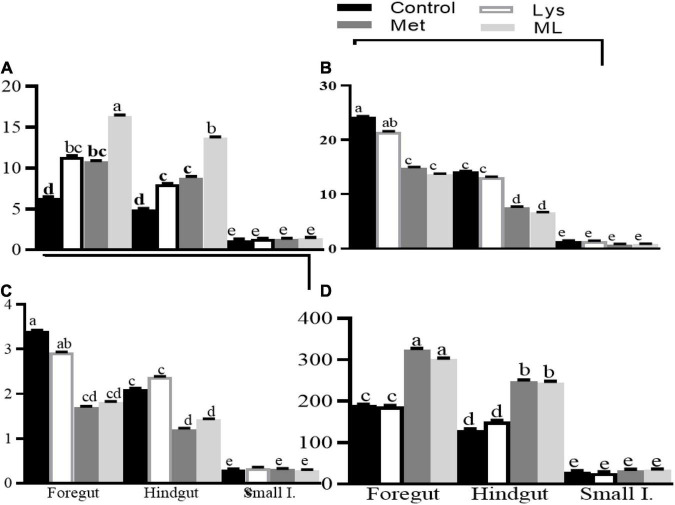
Least square and standard error of means (SEM) on microbial fermentation metabolites **(A)** ammonia production (mM), **(B)** dissolved hydrogen (dH_2_; μM), **(C)** dissolved methane (dCH_4_; mM), and **(D)** dissolved hydrogen sulfur (dH_2_S; mM) in different gut regions of goats in response to amino acid supplements. Different letters for the dietary treatments within each gut region indicate significant differences at *p* < 0.01.

Amino acid supplementation influenced ammonia and gaseous production, including dH_2_, dH_2_S, and dCH_4_, in different gut regions of goats (Table 4 and Figures 2, 3). The ammonia levels (+88 and 85%, respectively) were higher in the foregut and hindgut than in the small intestine, with the hindgut having less ammonia (−21%) than the foregut (Table 4 and Figure 2A). Foregut and hindgut had greater dH_2_ (+93 and 87%; *p* < 0.05), respectively, than in the small intestine with an apparently lower (−47.5%; *p* < 0.05) value in the contents of the hindgut vs. foregut (Table 4 and Figure 2B). In addition, the foregut and hindgut had greater dCH_4_ (+86 and 79%; *p* < 0.05) than in the small intestine and less (−30; *p* < 0.05) in the hindgut than the foregut (Table 4 and Figure 2C). Consistently, the foregut and hindgut had enhanced dH_2_S (+89 and 86%) levels than the small intestine and lower (−22%) dH_2_S levels in the hindgut than the foregut (Table 4 and Figure 2D). Goats fed ML had the highest ammonia compared with other treatments, whilst goats fed either Met alone or in combination had greater ammonia (*p* < 0.05) compared, with those in control (Table 4 and Figure 2E). In addition, goats fed either Met alone or in combination, reduced dH_2_, dCH_4_, while having greater dH_2_S production, than those in control or Lys (Table 4 and Figures 2F–H). It was consistent with higher ammonia in the contents of the foregut, followed by the hindgut in the amino acid supplements than in the control with ML having the highest ammonia levels (Table 4 and Figure 3A; *p* < 0.01). In addition, goats fed either Met alone or in combination, had reduced dH_2_, dCH_4_, while having increased dH_2_S (*p*0.05) than in control or Lys with a notably higher value in the foregut than in the hindgut and small intestine (Table 4 and Figures 3B–D; *p* < 0.01).

**TABLE 4:** Gas metabolites in gut regions of goats supplemented with amino acid.

Gut regions	Diet^2^	Items				
		pH	Ammonia (mM)	dH_2_^1^	dCH_4_	dH_2_S
Small intestine	Control	7.10	1.20^e^	1.40^e^	0.30^e^	29.9^e^
	Met	7.00	1.30^e^	0.77^e^	0.31^e^	33.0^e^
	Lys	6.05	1.31^e^	1.37^e^	0.34^e^	27.0^e^
	ML	7.09	1.41^e^	0.80^e^	0.28^e^	34.0^e^
Foregut	Control	6.87	6.37^d^	24.3^a^	3.41^a^	190.4^c^
	Met	7.20	10.8^bc^	14.9^c^	1.70^cd^	325.3^a^
	Lys	6.70	11.4^bc^	21.5^ab^	2.92^ab^	187.3^c^
	ML	6.90	16.4^a^	13.7^c^	1.81^cd^	301.8^a^
Hindgut	Control	6.85	4.94^d^	14.2^c^	2.11^c^	131.2^d^
	Met	6.98	8.85^c^	7.62^d^	1.21^d^	249.0^b^
	Lys	7.06	8.01^c^	13.1^c^	2.37^c^	151.0^d^
	ML	6.96	13.7^b^	6.63^d^	1.42^d^	245.8^b^
	SEM	0.24	0.12	0.1	0.02	2.59
*P-value*	Gut	0.2134	0.0302	0.0434	0.0302	0.0200
	Diet	0.1425	0.0300	0.0329	0.0400	0.0334
	Gut*Diet	0.1201	<0.0001	<0.0001	<0.0001	<0.0001

*^1^Dissolved hydrogen (μM), dissolved methane (mM), dissolved hydrogen sulfur (mM).*

*^2^Basic diet without supplementation (Control), control supplemented with methionine (Met), and control supplemented with lysine (Lys), control supplemented with methionine and lysine (ML). Results beard different letters indicate significant, while results beard same letters indicate not significant variation.*

### Association of Metabolome and Dissolved Gasses

There were positive correlations between dH_2_, dCH_4_ with ammonia, total VFA and molar percentages of propionate, isobutyrate, and isovalerate, and negative correlations with acetate, butyrate, and valerate, both in the foregut and hindgut, with no significant correlations in the small intestinal contents (Table 5). Furthermore, there was a strong positive correlation between dH_2_ and dCH_4_, while having a negative correlation with dH_2_S in the foregut and hindgut. Nevertheless, there was a negative correlation between dCH_4_ and dH_2_S in the foregut and hindgut. In addition, there was no significant correlation between dissolved gasses and fermentation metabolome in the contents of the small intestine.

**TABLE 5:** Correlation^1^ between dissolved hydrogen and fermentation metabolites in the gut regions of goats supplemented with amino acid.

Item	Dissolved H_2_			Dissolved CH_4_		
	Small I	Foregut	Hindgut	Small I	Foregut	Hindgut
Ammonia (mM)	0.10	0.90	0.65	0.09	0.82	0.67
Total volatile fatty acids (mM)	0.02	0.89	0.71	0.01	0.85	0.64
Acetate	0.03	0.82	0.62	0.00	0.92	0.72
Propionate	0.06	0.84	0.64	0.06	0.90	0.69
Butyrate	0.01	0.77	0.69	0.07	0.82	0.74
Valerate	0.04	0.73	0.71	0.11	0.65	0.69
Isobutyrate	0.03	0.82	0.68	0.03	0.78	0.58
Isovalerate	0.05	0.79	0.62	0.02	0.85	0.71
**Individual fatty acids (mol/100 mol)**						
Acetate	−0.08	−0.95	−0.71	−0.10	−0.84	−0.79
Propionate	0.13	0.82	0.69	0.06	0.79	0.59
Butyrate	−0.11	−0.61	−0.50	−0.12	−0.89	−0.62
Valerate	−0.09	−0.71	−0.64	−0.08	−0.75	−0.72
Isobutyrate	0.13	0.72	0.57	0.07	0.81	0.64
Isovalerate	0.17	0.89	0.74	0.13	0.73	0.66
Acetate to propionate ratio	−0.07	−0.83	−0.81	−0.10	−0.79	−0.70
**Dissolved gasses (mM)**						
Dissolved methane (dCH_4_)	0.04	0.93	0.81			
Dissolved hydrogen sulfur (dH_2_S)	−0.06	−0.95	−0.83	−0.05	−0.91	−0.74

*^1^Correlation coefficient values (r) with r > 0.44 for p < 0.1, r > 0.52, p < 0.05, r > 0.66 for p < 0.01, r > 0.79 for p < 0.001.*

### Microbiome

Amino acid supplementation had modulated gene copies of gut microbial ecosystems associated with particles and liquid fractions in goats (Table 6). The highest 16S rRNA gene copies of methanogens, bacteria, and 18S rRNA gene copies of fungi and protozoa (*p* < 0.01) were observed in the order of foregut > hindgut > small intestine. Additionally, 16S rRNA gene copies of starch (*Salmonella ruminantium*, *Prevotella ruminicola*, and *Ruminobacter amylophilus*) and fiber utilizing bacterial species (*Ruminococcus albus, Ruminococcus flavefaciens*, and *Fibrobacter succinogenes*) were also consistently greater in the foregut and hindgut than the small intestine. Goats fed amino acid supplements had increased 16S rRNA gene copies of bacteria, methanogens, and 18S rRNA of protozoa, fungi, and bacterial species (starch and fiber utilizing) (*p* < 0.05) than those in control. In addition, the interaction of gut regions with diet had modulated gene copies of microbiota (Table 6). In response to the amino acid supplements total bacteria, methanogens, protozoa, fungi, and functional bacterial species (*p* < 0.01) were increased in the foregut and hindgut compared with control.

**TABLE 6:** Microbiota^2^ in different gut regions of goats supplemented with amino acid.

Gut regions	Fraction	Diet^1^	Items	Total microbial population			Bacterial species					
			*Bacteria*	*Methanogens*	*Protozoa*	*Fungi*	*P. ruminicola*	*S. ruminantium*	*R. amylophilus*	*R. albus*	*R. flavefaciens*	*F. succinogenes*
Small intestine	Particle	Control	4.92^g^	4.01^f^	4.62^g^	3.29^g^	4.97^f^	4.39^f^	4.09	4.39^g^	4.10^g^	3.96^g^
		Met	5.15^g^	4.18^f^	4.83^g^	4.04^g^	4.59^f^	4.27^f^	3.99	4.49^g^	4.19^g^	4.06^g^
		Lys	5.01^g^	4.09^f^	4.60^g^	4.17^g^	4.92^f^	4.38^f^	3.96	4.45^g^	3.99^g^	3.89^g^
		ML	5.07^g^	4.06^f^	4.69^g^	4.89^g^	5.01^f^	4.52^f^	4.04	4.53^g^	4.08^g^	4.08^g^
	Liquid	Control	4.82^g^	3.93^f^	4.24^g^	3.02^g^	5.07^f^	4.36^f^	4.02	4.22^g^	4.03^g^	4.78^g^
		Met	5.05^g^	4.18^f^	4.40^g^	4.32^g^	4.79^f^	4.42^f^	3.47	4.39^g^	4.10^e^	3.99^g^
		Lys	5.50^g^	3.89^f^	3.97^g^	4.43^g^	4.84^f^	4.51^f^	3.40	4.28^e^	3.57^g^	4.92^g^
		ML	5.27^g^	4.16^f^	4.74^g^	4.27^g^	4.62^f^	4.39^f^	4.10	4.39^g^	4.01^g^	3.97^g^
Foregut	Particle	Control	10.6^b^	8.84^b^	9.06^b^	8.58^b^	9.21^c^	8.01^d^	9.01	10.9^b^	10.8^b^	10.1^b^
		Met	11.9^a^	9.97^a^	10.8^a^	9.78^a^	9.10^c^	9.63^c^	10.0	10.7^a^	11.1^a^	11.7^a^
		Lys	11.4^a^	9.67^a^	10.9^a^	9.88^a^	9.01^c^	9.16^c^	10.9	11.0^a^	10.7^a^	10.8^a^
		ML	12.2^a^	9.82^a^	11.3^a^	10.2^a^	10.6^b^	10.4^b^	10.2	10.9^a^	11.0^a^	10.6^a^
	Liquid	Control	8.07^d^	6.14^d^	6.99^e^	6.73^cd^	10.9^b^	10.0^b^	9.03	8.03^d^	8.02^d^	8.82^d^
		Met	9.70^c^	7.95^c^	8.52^c^	7.74^c^	11.0^a^	11.0^a^	10.3	9.89^c^	9.76^c^	9.78^c^
		Lys	9.87^c^	7.91^c^	8.67^c^	7.82^c^	10.8^a^	10.9^a^	10.6	9.91^c^	9.94^c^	9.86^c^
		ML	9.99^c^	8.02^bc^	9.04^b^	8.20^bc^	11.7^a^	11.2^a^	10.0	9.86^c^	9.84^c^	10.0^c^
Hindgut	Particle	Control	7.24^e^	6.19^d^	6.63^e^	6.01^e^	7.61^e^	7.89^e^	8.78	7.15^e^	7.01^e^	8.05^e^
		Met	9.59^c^	7.04^c^	7.88^d^	6.98^cd^	8.01^d^	8.87^d^	9.98	8.89^d^	8.19^d^	9.79^d^
		Lys	9.69^c^	7.14^c^	7.95^d^	7.01^cd^	8.02^d^	8.82^d^	9.89	8.97^d^	8.27^d^	9.78^d^
		ML	9.81^c^	7.10^c^	7.89^d^	6.81^cd^	8.09^d^	9.45^c^	9.97	9.09^d^	8.16^d^	9.69^d^
	Liquid	Control	6.04^f^	4.29^d^	4.13^g^	4.97^g^	8.11^d^	8.09^d^	8.89	5.09^f^	6.91^f^	6.19^f^
		Met	6.99^e^	5.82^e^	5.88^f^	5.98^f^	9.99^c^	9.01^c^	9.62	7.23^e^	7.89^e^	7.89^e^
		Lys	7.26^e^	5.94^e^	5.84^f^	5.85^f^	10.0^c^	9.32^c^	9.78	6.99^e^	7.97^e^	7.93^e^
		ML	7.01^e^	6.10^d^	5.73^f^	6.01^e^	9.97^c^	10.0^b^	9.80	7.09^e^	7.16^e^	7.95^e^
		SEM	0.20	0.32	0.06	0.03	0.10	0.02	0.36	0.03	1.20	0.02
*P-value*		Gut (G)	<0.0001	<0.0001	<0.0001	<0.0001	<0.0001	<0.0001	<0.0001	<0.0001	<0.0001	<0.0001
		Diet (D)	0.0303	0.0300	0.04102	0.0200	0.0223	0.0421	0.0311	0.0321	0.0400	0.0323
		Fraction	<0.0001	<0.0001	<0.0001	<0.0001	0.0193	0.0120	0.0244	<0.0001	<0.0001	<0.0001
		Gut*Diet	<0.0001	<0.0001	<0.0001	<0.0001	<0.0001	<0.0001	<0.0001	<0.0001	<0.0001	<0.0001
		Gut*F	<0.0001	<0.0001	<0.0001	<0.0001	0.0234	0.0310	0.0120	<0.0001	<0.0001	<0.0001
		Diet*F	<0.0001	<0.0001	<0.0001	<0.0001	<0.0001	<0.0001	<0.0001	<0.0001	<0.0001	<0.0001
		G*D*F	<0.0001	<0.0001	<0.0001	<0.0001	0.2102	0.3101	0.1901	<0.0001	<0.0001	<0.0001

*^1^Basic diet without supplementation (control), control supplemented with methionine (Met), and control supplemented with lysine (Lys), and control supplemented with methionine and lysine (ML).*

*^2^Microbiota log_10_ gene copy number per g of particle and liquid fractions of digesta in different gut regions of goats.*

*Results beard different letters indicate significant, while results beard same letters indicate not significant variation.*

Regardless of the amino acid supplemented, greater populations of methanogens, bacteria, fungi, protozoa, and fiber utilizing bacterial species (*R. albus, R. flavefaciens*, and *F. succinogenes*), while less *S. ruminantium, P. ruminicola* (*p* < 0.05) were observed in the particles, than liquid fractions. In addition, particles associated populations of the methanogens, bacteria, fungi, protozoa, and fiber utilizing bacterial species (*p* < 0.01) increased, both in the foregut and hindgut than liquid fractions. Nevertheless, populations of *S. ruminantium* and *P. ruminicola* were higher in the liquid than particles associated fractions, both in the foregut and hindgut. Consistently, goats in the amino acid supplements had increased populations of total microbiota, fiber utilizing bacterial species associated with particles vs. liquid fractions, both in the foregut and hindgut than in the control treatment with a notably highest value in the foregut. Moreover, the foregut and hindgut in the amino acids had increased total populations of bacteria, methanogens, protozoa, fungi, and fiber utilizing bacteria species (*p* < 0.01) associated with particles rather than liquid fractions in the control group. The highest values of these microbiotas were observed in the foregut than the hindgut, and the lowest in the small intestine (Table 6).

### Association of Gasses and Microbiome

Concentrations of microbial groups were in correlation with almost all fermentation metabolites, both in the foregut and hindgut (Table 7). Concentrations of total VFAs, dH_2,_ and ammonia, and molar proportions of acetate, isobutyrate, and isovalerate, were positively correlated with all microbial groups in the foregut and hindgut. On the other hand, the concentration of dH_2_S was positively correlated with the DNA concentrations of bacteria, protozoa, and fungi, and negatively correlated with methanogens. Inversely, dCH_4_ was negatively correlated with bacteria, protozoa, and fungi, but positively correlated with methanogens and fiber degrading bacterial species. Furthermore, the molar percentage of propionate was positively correlated with bacteria and fungi, while, negatively correlated with protozoa and methanogens, and fiber degrading bacterial species. On the other hand, molar percentages of butyrate and valerate were negatively correlated with concentrations of all microbial groups considered in the foregut and hindgut (Table 7).

**TABLE 7:** Correlation^1^ between concentrations of microbial groups and fermentation metabolites in the gut regions of goats supplemented with amino acid.

	Bacteria^1^		methanogen		protozoa		Fungi		*R. albus*		*R. flavefaciens*		*F. succinogenes*	
	Foregut	Hindgut	Foregut	Hindgut	Foregut	Hindgut	Foregut	Hindgut	Foregut	Hindgut	Foregut	Hindgut	Foregut	Hindgut
Protozoa	0.92	0.65	0.85	0.80			0.57	0.51	0.64	0.56	0.62	0.54	0.91	0.47
Fungi	0.78	0.73	0.78	0.51	0.54	0.54			0.73	0.57	0.87	0.63	0.82	0.62
Methanogen	0.89	0.70			0.78	0.65	0.86	0.75	0.67	0.51	0.71	0.69	0.73	0.51
*R. albus*	0.90	0.54	0.90	0.79	0.57	0.59	0.68	0.65			0.89	0.73	0.85	0.66
*R. flavefaciens*	0.76	0.59	0.83	0.64	0.75	0.66	0.85	0.74	0.85	0.67			0.89	0.57
*F. succinogenes*	0.86	0.60	0.89	0.74	0.87	0.49	0.79	0.63	0.78	0.53	0.96	0.67		
To VFA^2^	0.86	0.78	0.64	0.75	0.86	0.67	0.89	0.69	0.83	0.53	0.86	0.89	0.86	0.65
dH_2_^3^	0.75	0.63	0.74	0.64	0.59	0.86	0.79	0.73	0.69	0.45	0.58	0.86	0.75	0.68
dH_2_S	0.63	0.53	−0.53	−0.74	0.64	0.57	0.49	0.64	0.59	0.67	0.97	0.86	0.86	0.68
dCH_4_	−0.85	−0.64	0.58	0.47	−0.58	−0.68	−0.85	−0.64	−0.85	−0.75	−0.68	−0.67	−0.86	−0.63
Ammonia^4^	0.85	0.83	0.79	0.74	0.74	0.75	0.71	0.72	0.84	0.78	0.86	0.68	0.76	0.67
Acetate^5^	0.68	0.63	0.75	0.63	0.64	0.75	0.69	0.62	0.73	0.68	0.64	0.84	0.69	0.62
Propionate	0.65	0.68	−0.68	−0.83	−0.75	−0.58	0/74	0.72	−0.78	−0.97	−0.85	−0.69	−0.83	−0.68
Butyrate	−0.63	−0.54	−0.84	−0.64	−0.57	−0.75	−0.68	−0.72	−0.62	−0.61	−0.72	−0.75	−0.83	−0.69
Valerate	−0.27	−0.54	−0.49	−0.53	−0.67	−0.57	−0.49	−0.54	−0.56	−0.75	−0.74	−0.82	−0.75	−0.58
Isobutyrate	0.85	0.74	0.68	0.84	0.57	0.64	0.68	0.62	0.43	0.67	0.72	0.73	0.68	0.72
Isovalerate	0.74	0.63	0.58	0.53	0.50	0.78	0.72	0.52	0.78	0.47	0.68	0.59	0.69	0.58

*^1^Microbial concentrations in the gut regions were expressed as log_10_ transformed; correlation coefficient values (r) with r > 0.44 for p < 0.1, r > 0.52, p < 0.05, r > 0.66 for p < 0.01, r > 0.79 for p < 0.001. r-values < 0.1 is not presented in the table.*

*^2^Total volatile fatty acids (mM).*

*^3^Dissolved hydrogen (μM), dissolved methane (mM), dissolved hydrogen sulfur (mM).*

*^4^Ammonia (mM).*

*^5^Molar percentages of acetate, propionate, butyrate, valerate, isobutyrate, and isovalerate.*

## Discussion

We have proposed that sulfur-containing amino acids could redirect hydrogen toward an alternative sink (H_2_S) than methanogenesis and modulates metabolites and microbiota associated with particles and liquid fractions in the gut regions of goats. This was supported by a significant shift of these values both in the foregut and hindgut, with little effect on the small intestinal contents. This is likely because, the small intestine is predominated by enzymatic digestion, rather than microbial fermentation in the foregut and hindgut segments, implying less or no effect on fermentation products in the small intestine, in response to the amino acid supplements.

The influence of Met alone or in combination on the shift of fermentation metabolites such as dissolved gasses, VFAs, and the microbial community was visible, both in the foregut and hindgut, but, had little effect on the small intestine (Figure 3). This shows that the methane mitigating effects of these amino acid supplements are induced, not only by rumen fermentation modifications but also by hindgut fermentation changes. In this study, the decreased acetate to propionate ratio and CH_4_, both in the foregut and hindgut of goats fed, either Met alone or in combination, indicated the use of these supplements in mitigating methanogenesis which was consistent with decreased total gas and CH_4_ in methionine supplemented more than in the control group in an *in vitro* trial (Abbasi et al., 2019).

However, an increased propionate in goats fed Met alone or in combination, could be caused by the sulfur contained in the amino acids. This claim is supported by previous studies that highlighted the role of sulfur supplementation at different doses (1 to 2.5%) in increased propionate in the range of (1 to 10.9%) in ruminants (Bal and Ozturk, 2006; Promkot et al., 2007; Supapong and Cherdthong, 2020a,b). This observation was consistent with a previous study that reported goats feeding on high sulfur in corn gluten; (CG) reduced CH_4_ production and yield, and this was associated with decreased rumen liquid dH_2_ and dCH_4_, and increased dH_2_S, as compared with those fed low sulfur in corn meal (CM; Teklebrhan et al., 2020). In addition, the inclusion of 2% sulfur with 2.5% urea in the fermented total mixed ration (FTMR), improved digestibility, fermentation, microbial crude protein synthesis, and milk quality in dairy cows (Supapong and Cherdthong, 2020a,b), suggesting that sulfur redirects H_2_ toward energetically beneficial pathways for the animal against methanogenesis.

The dH_2_ plays a central role in regulating fermentation pathways; low dH_2_ stimulates the acetate production pathway, while high dH_2_ stimulates the propionate production pathway (Janssen, 2010). This was consistent with a positive correlation of dH_2_ with propionate proportion; and a negative correlation with acetate proportion in the rumen (Wang et al., 2016b,^2018^; Teklebrhan et al., 2020). Similarly, in the current study, we have observed a positive correlation of dH_2_ with propionate proportion and a negative correlation with acetate proportion, both in the foregut and hindgut. A shift in fermentation pathways affects CH_4_ production because, acetate biosynthesis is associated with net H_2_ release while, propionate formation is associated with reduced H_2_ formation (Janssen, 2010).

A recent *in vitro* study has reported a reduced H_2_ recovery in the methionine supplement than in the control (Hassan et al., 2021). This is in line with the current study, the decreasing H_2_ in the foregut and hindgut of goats fed Met alone or in combination might be due to the following main reasons: (1) Sulfur-containing amino acids such, as Met, may over compute methanogens for H_2_ because sulfur can be used as a potential H_2_ sink (Teklebrhan et al., 2020). (2) Increased free amino acids may increase deamination of the process with increased ammonia accumulation, implying less efficient amino acid utilization for microbial growth, resulting in reduced H_2_ release in the gut regions. (3) On the other hand, the increased dH_2_ in the foregut and hindgut of goats fed control treatment suggests a thermodynamically less efficient H_2_ consumption by methanogens, thus, stimulating the fermentation pathway that releases less H_2_ than more H_2_, per unit of glucose fermented in the gut regions. This was associated with increased propionate, over the acetate pathway in the foregut and hindgut of goats fed sulfur-containing amino acids, i.e., Met rather than control or Lys.

Sulfur-containing amino acids shifted H_2_ to a different hydrogen sink, increasing dH_2_S production rather than CH_4_ production. This occurs under standard gut conditions because sulfidogenic bacteria have a higher affinity for H_2_ utilization than methanogens, suggesting thermodynamically methanogenesis is less favored than sulfate reduction (Van Zijderveld et al., 2010; Judy et al., 2019; Lan and Yang, 2019; Teklebrhan et al., 2020). Because sulfur-reducing microbes use sulfur as an electron sinker or acceptor, and the reduced forms of sulfur H_2_S is a metabolic end-product of fermentation from these microorganisms and noticed in the current study. It is related to the reduced forms of sulfur observed in an earlier study (Gould, 2000). This is supported by our findings of increased H_2_S than methanogenesis in the foregut and hindgut; in response to sulfur-containing amino acid supplementation. Similarly, methanogenesis was reduced in goats fed; a high sulfur-containing diet of CG vs. CM (Teklebrhan et al., 2020), reporting increased H_2_S vis-à-vis CH_4_ in the rumen liquid. In addition, an *in vitro* culturing system was found to reduce CH_4_ in methionine addition more than in the control group (Abbasi et al., 2019; Hassan et al., 2021). This observation was supported by a negative correlation between concentrations of methanogens, dCH_4,_ and dH_2_ with dH_2_S, both in the foregut and hindgut contents in the current study.

The gut microbiome is a complex ecosystem of bacteria, methanogens, archaea, fungi, and protozoa, and bacteriophages, which interact with each other and their host (Goodman and Gordon, 2010; Minot et al., 2011). Understanding of their composition, association with their metabolome, and ecological role gives insight into how to improve nutrient utilization efficiencies and health and reduce the carbon footprint of ruminants. The significant associations with microbes and their metabolites suggest that the microbiome plays a significant role in the fermentation of amino acids to ammonia, VFA, CO_2_, CH_4_, and H_2_ for microbial protein synthesis in the gut regions of ruminants. In the current study, in response to amino acid supplement, we observed increased copies of 16S rRNA methanogens, bacteria, and 18S rRNA protozoa, fungi, and fiber utilizing bacteria species in the particles associated with liquid fractions in the foregut and hindgut, with a notable greatest value in the foregut. In addition, regardless of the amino acid supplements and sampling fraction, gut regions significantly increased total microbial populations of methanogens, bacteria, protozoa, fungi, as well as starch and fiber, utilizing bacteria species in the order of foregut > hindgut > small intestine. The decreased copies of these microbiomes could be caused by less digestible matters and a slower fermentation rate in the small intestine than in the foregut and hindgut regions. Similarly, previous studies have reported lower total gene copies of protozoa, methanogens, bacteria, and fiber degrading bacterial species (*F. succinogenes*, *R. albus*, and *R. flavefaciens*) in the cecal and small intestinal contents vs. ruminal contents (Shinkai and Kobayashi, 2007; Frey et al., 2010; Popova et al., 2013). In addition, increasing copies of these genes were documented in the contents of the rumen, rather than in small and large intestines (Zeng et al., 2015, 2017). These lower populations of these microbiomes in the hindgut and small intestine might be, due to the lower rate and activity of fiber degrading enzymes, than in the foregut. This was in agreement with an earlier study by Bauer et al. (2004) that reported less cellulolytic enzyme activities in the contents of the small and large intestines. This might be related to the correlations observed between microbiota and microbial fermentation metabolites, in the foregut and hindgut of goats, which suggests increased gene copies of total microbiota and functional bacterial species in these gut regions in the current study. For example, the increased populations of fiber degrading bacterial species, both in the foregut and hindgut suggests these microbes are useful for fiber degradation, mainly; in the foregut, and fully undegradable fractions in the hindgut. This is supported by significant correlations between the concentration of metabolome and the associated proliferation of fiber degrading bacterial species, both in the foregut and hindgut, as observed in the current study.

Several studies have described that dietary supplements can influence microbial populations and functional bacterial species in ruminal contents (Thoetkiattikul et al., 2013; Pan et al., 2017; Xue et al., 2018; Teklebrhan et al., 2020). Nevertheless, only a few studies have used amino acid supplements for microbial quantification studies and, most of them are *in vitro* highlighting increased microbial copy numbers, using methionine than in the control groups. The authors reported that methionine addition can increase the protozoa and *R. albus* population, both in particles associated and liquid fractions (Abbasi et al., 2019; Hassan et al., 2021). Moreover, Martin et al. (2013) described that methionine supplementation, either alone or in combination, can increase fibrinolytic bacterial species including *R. flavefaciens, F. succinogenes*, and *R. albus*. Earlier studies have also reported that ruminal bacteria such as *R. amylophilus* and *Prevotella* spp. are actively engaged in protein degradation (Blackburn and Hobson, 1962; Wallace, 1994). These findings were consistent with higher populations of total bacteria, protozoa, methanogens, fungi, and bacterial species in goats fed on the amino acid supplements, rather than in the control of the current study. The increase of these microbiomes in the foregut and hindgut might be caused by the increase of free amino acid availability to microbes, suggesting that elevating microbial protein synthesis, which is essential for microbial growth and associated with increasing gene copies in the foregut, as well as hindgut. Interestingly, nitrogen losses in the feces and urine were similar to the amino acid supplements vs. control (Zhu et al., 2018), implying that free amino acid supplements are not always causing nitrogen waste and excretion, if they are with low total dietary protein (12.69% CP), as in the current study. It is consistent with less nitrogen excretion in the feces and urine, when animals are fed on rations containing low protein 12–18% CP and has no adverse effect on the energy balance of ruminants (Broderick et al., 2008; Agle et al., 2010; Erickson and Klopfenstein, 2010).

Most of the microbial quantification studies use rumen liquid samples. This technique may underestimate the number of microbes that remained attached to fiber particles that could have significantly affected the total microbiota inhabiting the ruminant gut. Similarly, earlier studies have suggested that microorganisms attaching to undigested feed particles account for a significant proportion of total ruminal microorganisms (Merry and McAllan, 1983; Craig et al., 1987a,b) stated that particles associated with microbial organic matter (OM), accounted for 70 to 80% of the total microbial mass in the rumen. A previous study has assessed microbes in the rumen liquid or particles associated fractions (Mullins et al., 2013), reporting that total bacteria, fungi, *F. succinogenes*, and *R. albus* populations were higher in the particles associated fractions than in liquid (Mullins et al., 2013). Likewise, in this study, we have investigated the differences in the microbiota of goats fed amino acid in the foregut, small intestine, and hindgut in the particles associated and liquid fractions. The total populations of microbiota and fiber degrading bacterial species of *R*. *albus*, *F. succinogenes*, and, *R. flavefaciens* were increased in the particles associated, with liquid fractions in the current study. This suggests these bacterial species are predominantly engaged in degrading and fermenting plant fibers, which is in accordance with Flint et al. (2008) and Biddle et al. (2013).

## Conclusion

The hypothesis of this study is that sulfur amino acids could shift hydrogen toward an alternative sink supported by increased H_2_S instead of methanogenesis and changed fermentation and microbiota, associated with particles and liquid fractions, both in the foregut and hindgut of goats. Goats fed on Met and Lys either alone or in combination had increased 16S rRNA gene copies of total bacteria, methanogens, and 18S rRNA of protozoa, fungi, and fiber; utilizing bacterial species associated with particles than liquid fractions of those in control. In addition, amino acid supplements increased total bacteria, methanogens, protozoa and fungi populations, fiber, and starch utilizing bacterial species both in the foregut and hindgut compared with the control group. This study suggests that sulfur-containing amino acids shift hydrogen to an alternative hydrogen sink, i.e., H_2_S, over methanogenesis and modified gut fermentation metabolites, increasing particle-associated microbiota than liquid both in the foregut and hindgut. This study gives insights into the use of sulfur-containing amino acids, as an alternative dietary mitigation strategy of methanogenesis in ruminants, and it underscores the need for related further research on sulfur amino acids, as a potential sink of hydrogen.

## Data Availability Statement

The raw data supporting the conclusions of this article will be made available by the authors, without undue reservation.

## Ethics Statement

The animal study was reviewed and approved by the experimental protocols used in this trial endorsed by the animal care and use committee of the Institute of Subtropical Agriculture, Chinese Academy of Sciences and strictly followed the guidelines for animal welfare established by the committee.

## Author Contributions

TT designed, conducted, and analyzed the experiment and wrote the manuscript. ZT revised and edited the manuscript. Both authors read and approved the manuscript for submission.

## Conflict of Interest

The authors declare that the research was conducted in the absence of any commercial or financial relationships that could be construed as a potential conflict of interest.

## Publisher’s Note

All claims expressed in this article are solely those of the authors and do not necessarily represent those of their affiliated organizations, or those of the publisher, the editors and the reviewers. Any product that may be evaluated in this article, or claim that may be made by its manufacturer, is not guaranteed or endorsed by the publisher.
